# Modification of Peyton’s four-step approach for small group teaching – a descriptive study

**DOI:** 10.1186/1472-6920-14-68

**Published:** 2014-04-02

**Authors:** Christoph Nikendei, Julia Huber, Jan Stiepak, Daniel Huhn, Jan Lauter, Wolfgang Herzog, Jana Jünger, Markus Krautter

**Affiliations:** 1 Department of General Internal and Psychosomatic Medicine, University of Heidelberg, Heidelberg, Germany; 2 Department of Cardiology, Angiology, Pneumology, University of Heidelberg, Heidelberg, Germany; 3 Department of Nephrology, University of Heidelberg, Heidelberg, Germany

**Keywords:** Undergraduate medical education, Clinical skills, Catheter insertion, Simulation, Instructional approach, Peyton, Small group teaching

## Abstract

**Background:**

Skills-lab training as a methodological teaching approach is nowadays part of the training programs of almost all medical faculties. Specific ingredients have been shown to contribute to a successful learning experience in skills-labs. Although it is undoubted that the instructional approach used to introduce novel clinical technical skills to learners has a decisive impact on subsequent skills performance, as yet, little is known about differential effects of varying instructional methods. An instructional approach that is becoming increasingly prevalent in medical education is “Peyton’s Four-Step Approach”. As Peyton’s Four Step Approach was designed for a 1:1 teacher : student ratio, the aim of the present study was to develop and evaluate a modified Peyton’s Approach for small group teaching.

**Methods:**

The modified Peyton’s Approach was applied in three skills-lab training sessions on IV catheter insertion, each with three first- or second year medical students (n = 9), delivered by three different skills-lab teachers. The presented descriptive study investigated the practicability and subjective impressions of skills-lab trainees and tutors. Skills-lab sessions were evaluated by trainees’ self-assessment, expert ratings, and qualitative analysis of semi-standardized interviews conducted with trainees and tutors.

**Results:**

The model was well accepted by trainees, and was rated as easy to realize, resulting in a good flow of teaching and success in attracting trainee’s attention when observed by expert raters. Qualitative semi-standardized interviews performed with all of the trainees and tutors revealed that trainees valued repeated observation, instruction of trainees and the opportunity for independent performance, while tutors stressed that trainees were highly concentrated throughout the training and that they perceived repeated observation to be a valuable preparation for their own performance.

**Conclusion:**

The modified Peyton’s Approach to instruct small groups of students in skills-lab training sessions has revealed to be practicable, well accepted by trainees, and easy for tutors to realize. Further research should address the realization of the model in larger skills-lab training groups.

## Background

Skills-lab training as a methodological teaching approach is nowadays part of the training programs of almost all medical faculties. Skills-labs offer a protected, “mistake-forgiving” training environment [1] that allows students to practice procedures on mannequins, with standardized patients or with each other prior to performing procedural skills on real patients [2-4]. Skills-lab training has been shown to improve procedural skills both in novices and experts [5-8]. This applies to complex surgical skills [8] as well as basic clinical skills performed by medical students [9]. Furthermore, there seems to be evidence that simulation-based medical education (SBME) positively influences the outcome in the clinical setting [10]. Specific ingredients have been shown to contribute to a successful learning experience in skills-labs, such as pre-defined learning goals and curriculum integration, validity of the simulated scenarios [4,5], sustained deliberate practice [11] and feedback [12,13]. Although it is undoubted that the instructional approach used to introduce novel clinical technical skills to learners also has a decisive impact on subsequent skills performance [9,14], as yet, little is known about differential effects of varying instructional methods.

A variety of instructional approaches that include multiple steps to convey clinical technical skills to learners have been described in the literature [15,16]. A model that is becoming increasingly prevalent in medical education is “Peyton’s Four-Step Approach” [17-19], which – since the year 2000 – has also represented the standard instruction within training courses of the European Society of Cardiology (ESC) [20-23]. Peyton’s Four-Step Approach consists of four clearly defined instructional steps:

1. The teacher demonstrates the skill at his normal pace without any comments (“Demonstration”)

2. The teacher repeats the procedure, this time describing all necessary sub-steps (“Deconstruction”)

3. The student has to explain each sub-step while the teacher follows the student’s instructions (“Comprehension”)

4. The student performs the complete skill himself on his own (“Performance”).

Recently, Krautter et al. [14] were able to prove the effectiveness of Peyton’s Four-Step Approach for the teaching of gastric tube insertion using a mannequin. In a randomized controlled trial, volunteer second- and third-year medical students were randomly assigned to an intervention group receiving instruction according to Peyton’s Four-Step Approach or to a control group receiving standard instruction encompassing two demonstrations and a description of sub-steps. Following each of the two forms of instruction, participants’ first independent gastric tube insertions were video-recorded and scored by two independent video assessors. Results showed that the groups did not differ in terms of correct stepwise performance of the procedure as assessed by a binary checklist. However, ratings based on global rating scales assessing professionalism and accompanying patient-doctor communication proved to be significantly better in the intervention group. The authors assumed that these results could be mainly attributed to the 3^rd^ Step of Peyton’s Four-Step Approach, which involves mental representation of, but not actual, body movement [14]. Indeed, in a further study [24], Peyton’s Step 3 proved to be the most relevant part within Peyton’s Four-Step Approach. When the authors replaced Peyton’s Step 3 with a mere repetition of Step 2, Step 3 exceeded the benefit of a mere repetition of skills demonstration (repetition of Step 2).

As a limitation, however, Peyton’s Four-Step Approach was designed for teaching in a student-teacher ratio of 1:1, and not for small group teaching. This might lead to problems, as a student-teacher ratio of 1:1 does not reflect prevalent skills-lab training conditions. Generally, groups of medical students receiving skills-lab training consist of five to eight trainees or even more [4,25-28]. Therefore, the aim of the present study was to develop and evaluate a modified Peyton’s Approach for small group teaching. Research questions asked (i) whether the model of an adaptation of Peyton’s Four-Step Approach for small group teaching is practicable in terms of complexity, (ii) how the didactic value of the model is perceived by trainees and tutors, and (iii) how the professional realization of the model by skills-lab tutors is rated by external observers. We hypothesized that the model of a modified Peyton’s Approach for small group teaching would be practicable and well accepted by trainees and skills-lab trainers and could be easily realized by skills-lab tutors.

## Methods

### Development of the teaching model – expert group

Possible modifications of Peyton’s Four-Step Approach [19] for small group teaching were developed by an expert group (n = 4; one female; three male; mean age 37.0 ± 4.6 years; all of whom were certified skills-lab teachers (e.g. MME) with long-standing medical education and medical education research experience). The aim was to achieve an adaptation of the model that involves all students and preserves Step 3 of Peyton’s Four-Step Approach, as this step seems to be the crucial part of the didactic method [24]. Furthermore, every student should be able to perform the skill and receive feedback at least once. Advantages and disadvantages of modified models were discussed among these experts within a focus group approach. Finally, the favorite model was clearly defined and affixed. This modified Peyton’s Approach consists of the following parts detailed below:

A *– Demonstration and Deconstruction*

• The Tutor performs Steps 1 and 2 of Peyton’s Four-Step Approach to all Trainees

B *– Comprehension, Tutor’s Performance and Observation*

• The Tutor performs Step 3 following the instructions of Trainee 1, while all other Trainees are observing

C *– Comprehension, Trainee’s Performance and Observation*

• Trainee 1 performs Step 3 following instructions of Trainee 2, while the other Trainees are observing

D *– Tutor and Peer Feedback*

• Trainee 1 receives feedback by Peer Trainees, followed by Tutor feedback

E *– Circulation*

• Parts C and D are repeated in turn until the last Trainee has performed Step 3 following the instructions of a Trainee

F *– Completion and Conclusion*

• Finally, the last Trainee performs Step 4, followed by Peer and Tutor feedback.

The described model allows each of the trainees to perform the respective skill at least once. Part E (Circulation) leads to a combination of Steps 3 and 4, meaning that all of the trainees (except the last trainee) perform the task under verbal instructions of a peer trainee. Attentive peer observation followed by peer feedback is implemented in order to maintain the attention of all participating trainees and to draw benefit from the active performances of peer students.

### Study design

The presented descriptive study investigated the practicability and subjective impressions of skills-lab trainees and tutors during a skills-lab small group teaching session applying a modified Peyton’s Approach. IV catheter insertion was selected as the clinical task as this skill represents a pivotal routine procedure in internal medicine [29,30]. The study was conducted over a period of one week alongside the regular curriculum at our faculty.

Ethical principles according to the World Medical Association Declaration of Helsinki Ethical Principles for Medical Research Involving Human Subjects of 2008 were adhered to. The study protocol was approved by the ethics committee of the University of Heidelberg, decision number S-211/2009. Refusal to participate had no impact on the subsequent evaluations or other assessments in the curriculum.

### Evaluated skills-lab session integrating the modified Peyton’s Approach for small group teaching

#### Assessed skills-lab session

In order to evaluate the modified Peyton’s Approach to small group teaching, we performed three skills-lab sessions, each with three first- or second-year medical students (n = 9), delivered by three different skills-lab teachers. Within skills-lab training sessions, the adaptation of Peyton’s Four-Step Approach for small group teaching as described above was applied under supervision of one of the authors (C.N.) to monitor adherence to instructions.

#### Skills-lab trainees

Trainees in their first and second year of medical training were invited via advertisements to participate in the study and received a medical science book with a value of €20 as compensation. A detailed sample description is presented in the results section.

#### Skills-lab teachers

All of the skills-lab sessions were conducted by one of three skills-lab trainers (n = 3). All skills-lab trainers were male, with a mean age of 36.0 ± 5.0 years. They were all internal medicine consultants with considerable experience in delivering skills-lab and emergency training. Trainers were instructed by the expert team on how to realize the modified Peyton’s Approach for small group teaching.

### Pre-post self-efficacy ratings and questionnaire evaluation of skills-lab training by trainees

#### Pre-post self-efficacy ratings

Self-efficacy ratings were performed by all trainees pre- and post-skills-lab training. The questionnaire contained five items referring to a) knowledge of anatomical structures required for IV catheter insertion, b) knowledge of the materials required to insert an IV catheter, c) knowledge of the steps involved in placing an IV catheter, d) competence in inserting an IV catheter in a mannequin, and e) competence in inserting an IV catheter in a patient. These items were rated on a six-point Likert scale (1 = “I completely agree” to 6 = “I completely disagree”).

#### Evaluation of skills-lab sessions

Skills-lab training sessions were evaluated by trainees post-intervention in order to assess the students’ acceptance of the teaching model. Nine statements about the teaching modalities were rated on a six-point Likert scale (1 = “I completely agree” to 6 = “I completely disagree”). Items are shown in Table 1.

**Table 1:** Acceptance ratings of skills-lab training session provided by trainees (n = 9)

**Item**	**M**	**SD**
I have learned a lot during the training session	1.3	± 0.5
I was continuously alert during the training	1.3	± 0.5
The repeated observation of the procedure was helpful	1.1	± 0.3
The independent performance of the procedure was helpful	1.1	± 0.3
There were too few repeated observations of the procedure	4.7	± 1.3
There were too many repeated observations of the procedure	5.0	± 1.3
There were too few independent performances	2.9	± 1.4
Commenting on and instructing the procedure was helpful	1.0	± 0.0
Having finished the training, I feel well prepared for practicing the procedure independently	1.6	± 0.9
Having finished the training, I already felt secure in performing the procedure	2.9	± 1.3

Six-point Likert scale (1 = I fully agree; 6 = I fully disagree); mean and SD.

### Expert ratings

Two independent observers (n = 2; both male, mean age 31.5 ± 0.71 years) followed all of the teaching sessions. The expert raters were blinded to the study design as well as to the research question. Raters were asked to make notes relating to four didactical aspects while they were watching the teaching sessions and finally to rate these four aspects on a visual analogue scale (VAS) from 0 to 100 (0 = “I fully disagree” to 100 = “I fully agree”) for each skills-lab session by marking a point on a line. Didactical aspects were as follows:

1) *trainees’ comprehension of the didactic approach*

Further details for expert raters: Trainees are able to follow the training easily; are aware of what is demanded of them; can follow the instructions; do not become irritated; get involved in the method easily; don’t have to ask to clarify their understanding.

2) *trainees’ focus of attention*

Further details for expert raters: Trainees follow the instruction in a focused manner, don’t digress; stick to the point; don’t interrupt the training, don’t hinder the procedure.

3) *tutors’ self-assuredness*

Further details for expert raters: The trainer acts confidently and competently; is able to control the training session and maintain the concentration of the trainees; structures the training session; there are no obvious irritations or uncertainties.

4) *flow of the training*

Further details for expert raters: The training session runs fluidly and flawlessly; there are no times of idle state, no interruptions or irritations of the group; the instructions of the participants follow one after the other without further breaks.

### Time needed for skills-lab sessions

The amount of time needed for skills-lab sessions was recorded by the attendant supervisor, who was present to monitor adherence to instructions (C.N.).

### Qualitative analysis of semi-standardized interviews conducted with trainees and tutors

According to the main items of the COREQ checklist [31], in the following, we provide further information about the interview procedure. Skills-lab training was followed by semi-standardized interviews, which were held with all of the trainees (n = 9) and skills-lab trainers (n = 3). The individual face-to-face interviews were conducted in a semi-standardized manner [32-34] containing the main open-ended questions, followed by encouraging questions and clarifying questions if required. They were conducted by one trained interviewer (male, 32 years of age, psychologist and research assistant), following the semi-standardised interview manual, and the dialogue was audio-taped. The interviewer had been trained and was supervised by an experienced tutor and other colleagues who had conducted similar studies. He probed for more details and specific examples when necessary. Interviews lasted for approximately ten minutes each. The leading questions according to Helfferich [35] were:

•What are your impressions concerning the training session “Insertion of an IV catheter”, in which you just took part?

•What are your positive impressions?

•What are your negative impressions?

### Statistical analyses of questionnaires and qualitative analyses of semi-standardized interviews

Descriptive quantitative data are presented as means ± standard deviation. For pre-post self-efficacy ratings, MWU-tests for dependent samples were conducted. Inter-rater reliability of expert ratings was calculated as Pearson product–moment correlation coefficient. Regarding qualitative data, audio files of the 12 interviews were transcribed verbatim. The conducted content analysis followed the principles of grounded theory [36]. First, we conducted an open coding of all of the nine interviews of trainees and 3 interviews of skills-lab trainers in order to search for recurring topics. In detail, single or few sentences were identified as a code, representing the most elemental unit of meaning [36]. Next, the codes were summarised into relevant themes for each participant, using the software MaxQDA (2010 version, VERBI GmbH, Berlin). As themes were recurrent among different participants, themes were then compared and adapted, until a number of relevant themes for all trainees and all skills-lab trainers could be defined. The assignment of respective codes to specific themes was conducted by two independent analysers and subsequently discussed to reach consensus and, if required, adjusted. In the final step, themes were consolidated into four relevant categories.

## Results

### Sample description

Trainees (n =9; four female, five male; mean age 21.7 ± 3.6 years) in their first and second year of medical training participated in the study. One female participant had successfully completed medical training as a nurse prior to studying medicine. One male participant had successfully completed non-medical studies of biophysics and construction engineering prior to studying medicine. All participants were right-handed and had no prior experience in IV catheter insertion.

### Pre-post self-efficacy ratings and questionnaire evaluation of skills-lab training by trainees

#### Pre-post self-efficacy ratings

Comparison of pre (3.7 ± 1.5) and post (1.6 ± 0.5) self-efficacy ratings (1 = “I completely agree” to 6 = “I completely disagree) of participating trainees turned out to be significant (p < .001).

#### Evaluation of skills-lab sessions

Evaluations of the skills-lab training sessions on IV catheter insertion by participating trainees are displayed in Table 1.

### Expert ratings

Results of the expert rating regarding trainees’ comprehension of the didactic approach, trainees’ focus of attention, tutors’ confidence, and flow of the training is shown in Table 2. Inter-rater reliability of expert ratings was r = .87.

**Table 2:** Ratings of skills-lab training sessions by independent experts (n = 2)

**Item**	**M**	**SD**
Trainees’ comprehension of the didactic approach	93	± 3
Trainees’ focus of attention	96	± 4
Tutors’ self-assuredness	90	± 7
Flow of the training	92	± 4

Visual analogue scale (VAS) from 0 to 100 (0 = “I fully disagree” to 100 = “I fully agree”); mean and SD.

### Time needed for skills-lab sessions

The mean time needed for one skills-lab session encompassing a total of six IV catheter insertions was 37.5 ± 1.8 min.

### Qualitative analysis of semi-standardized interviews conducted with trainees and tutors

#### Themes and categories resulting from interviews with trainees and skills-lab tutors

Over the course of the interviews of trainees and skills-lab teachers saturation was reached. With regard to the qualitative analysis of the interview transcripts, 84 single statements of trainees and 22 single statements of skills-lab tutors were identified. From these codes, 14 themes clustered to 11 main categories for trainee interviews and ten themes clustered to eight main categories for tutor interviews were derived separately for both groups.

#### Definition of themes and categories resulting from interviews with trainees

A) Methodological approach: The methodological aspects which were implemented in the training were central to the students’ statements.

• Learning through observation and action: The students appreciated the opportunity to observe the action steps and subsequently perform them themselves. One student referred to the difficulty of his/her own practical performance.

• Learning through repetition: The students appreciated the multiple repetitions of the individual steps.

• Learning through teaching: The students experienced the switch into the role of teacher, in which they were able to instruct another student in the performance, as valuable.

• Insufficient number of practical performances: Several students wished to carry out more practical performances themselves.

B) Involvement: The students appreciated the involvement and participation of all participants in the whole training. In this respect, they addressed visual, auditory, motor and communicative learning types. Attention was maintained throughout the duration of the training.

C) Learning atmosphere and general conditions: The general conditions were perceived as good, and the learning atmosphere was described as personal.

D) General impression: The students evaluated the training as good and useful; one student underlined the superiority of this training over another learning method.

E) Time expenditure: The time expenditure was rated as low by the students.

F) Subjectively experienced learning success: The students emphasized the learning success that was achieved through the training. They reported that they were quickly able to commit the individual action steps to memory, to learn them and to practice them.

G) Use of the mannequin and anticipated transfer to the clinic: The students questioned the possibility to transfer the training to practice. The difference between the activity on the mannequin and the practical performance on a patient was emphasized and possible problems in the clinic were highlighted. One student deemed the training to be helpful in order to develop an impression of the practical activity.

H) Teaching in the small group: The students evaluated the small group size as positive, enabling personal conversations and a high involvement of all students.

I) Sequence of the training: The students described the sequence of the training as good. It was possible for them to think for themselves about the sequence of the individual steps. They were then able to use the theoretical knowledge in order to guide themselves in their own practical performance.

J) Precision of the training: The precision of the performance of the action steps was on the one hand praised. On the other hand, it was established that the performance on the mannequin only partially depicts reality.

K) Tutors: The students appreciated the instruction by an experienced physician in contrast to teaching by student tutors.

Codes related to categories A – K are displayed in Table 3.

**Table 3:** Codes related to themes and categories derived from qualitative analyses of semi-standardized interviews conducted with all trainees (n = 9); relevant codes within the quotations are bold

**Categories and themes**	**Codes**
*A.* Methodological approach	
• Learning through observation and action	*“So, what was actually really good was that you explained it to yourself first,* ***and then tried it out****, meaning that, as it were, you were again theoretically reminded of the individual action steps before performing them yourself.”*
• Learning through repetition	*“I found* ***it*** *fairly clear, also because the steps were constantly repeated just as much.”*
	*“And* ***because*** *when you’ve then heard it relatively often, it gradually gets better and better, each time you somehow pick more of it up.”*
• Learning through teaching	*“****Yes, I also found it good that you should formulate it yourself, so you instruct somebody to do it*** *and then you get to practice it again yourself, because then, in a way, you could simply give yourself these instructions in your head.”*
• Insufficient number of practical performances	*“I would have maybe performed it* ***once*** *or twice more, but of course there isn’t enough time for that.”*
*B.* Involvement	*“The fact that everyone is always integrated in some way, so everyone has to be attentive the whole time.”*
*C.* Learning atmosphere and general conditions	*“I found it very good* ***that it was such a personal conversation*** *between the trainer and the trainee, because, well there were four of us.”*
*D.* General impressions	*“Well, in the basics of medical clerkship, I had tried out a different principle, but I found this much better.”*
*E.* Time expenditure	*“So in itself I found it really good that with so little time expenditure you get something so important shown to you.”*
F. Subjectively experienced learning success	*“So, one of the best things was that after he had performed it twice, the second time with an explanation, that on the 3rd and 4th time somebody explained it while another person performed it.* ***And in that way it really did stick****.”*
*G.* Use of the mannequin and anticipated transfer to the clinic	“*It was good practicing it on a doll like that, that you already got a bit of an idea.”*
H. Teaching in the small group	*“And also in the small group it was very effective, very positive, if one could set the framework with 3 or 4 people, very good.”*
*I.* Sequence of the training	*“I also liked the fact that you* ***first watched the whole thing*** *without it being commented on,* ***and then*** *it was commented on in individual steps.”*
*J.* Precision of the training	*“Because above all, so for me, what I have seen elsewhere is that you completed the things you needed and then went to the patient, and for example almost placed no value on disinfecting and instead briefly sprayed, wiped over it, and then sprayed again and then went right in with the needle. And here, you waited and unpacked the things and everything, and then the disinfectant had really taken effect.”*
*K.* Tutors	*“Being instructed by somebody with experience, so in this case the senior physician is really clinically oriented.”*

#### Definition of themes and categories resulting from interviews with skills-lab tutors

A) Methodological approach: The methodological aspects that were implemented in the training were central to the tutors’ statements.

• Learning through observation: The tutors did not deem the time periods in which students were observing the performances and were not active to be problematic.

• Learning through repetition: The possibility to observe the steps repeatedly was judged to be informative.

• Learning through teaching and being instructed: One of the tutors felt that it appeared to be problematic for the students to be instructed by another student and in this respect to not anticipate the instructions based on their own prior knowledge due to the previous observation.

B) Involvement: The tutors emphasized the high attention, concentration and motivation, as well as the great interest of the participants across the whole training.

C) Learning atmosphere: The tutors appreciated the students’ constructive manner of dealing with one another, in particular with regard to mutual feedback.

D) General impressions: At the beginning of the training, the tutors were uncertain about its success; however, at the end of the training they showed themselves to be impressed by the appeal of the training.

E) Time expenditure: One of the tutors judged the time expenditure of the training as high.

F) Students’ performance: The tutors evaluated the students’ performance as good.

G) Group size and group processes: The tutors experienced the group size as suitable. The students worked together on their goals in an attentive manner, enabling a high level of group cohesion to be achieved.

H) Transfer: The training was seen as an important preparation of the students for the clinic.

Codes related to categories A – H are displayed in Table 4.

**Table 4:** Codes related to themes and categories derived from qualitative analyses of semi-standardized interviews conducted with skills-lab tutors (n = 3); relevant codes within the quotations are bold

**Categories and themes**	**Codes**
*A.* Methodological approach	
• Learning through observation	*“But during the whole thing, it became relaxed because you then noticed, ok, you can give those who aren’t doing anything right now some additional task in the sense of ‘observe and then at the end give some kind of feedback about what the two people performing might have missed’.”*
• Learning through repetition	*“I think, through the fact that you see it multiple times, and indeed even more often than if you only do it in 1:1 teaching, that is maybe a positive aspect.”*
• Learning through teaching and being instructed	*“First, the person who was doing it at the time and who was having it explained to him/her sometimes had problems, in quotes, actually “playing stupid”, as though he doesn’t know about anything, but he’d just seen it, and there, I think people tend to have problems playing stupid and not practically already doing the insertion before it has been explained to them.”*
*B.* Involvement	*“Yes, but with the group size it was now really the case that* ***the participants were always attentive, they didn’t somehow switch off from time to time****.”*
*C.* Learning atmosphere	*“So I found that they dealt with each other in a very constructive way.”*
*D.* General impressions	*“So of course, it you’re trying something out for the first time, you’re always a bit curious about how it will go.”*
*E.* Time expenditure	*“Is still a method that is simply time-consuming.”*
*F.* Students’ performance	*“And I had the impression that, IV catheter insertion is indeed difficult, but through the fact that the specific instructions were made according to Peyton, that they were very well prepared at the point when they had to insert the IV catheter themselves.”*
*G.* Group size and group processes	*“So what I liked, my experience was that it was a very concentrated group and it was like working together on a project, that people weren’t just looking out for themselves and only interested in themselves, but that everyone was very solicitous with each other and I found that a great kind of group cohesion arose.”*
*H.* Transfer	*“Was still an important preparation for the students.”*

## Discussion

To our knowledge, the current study is the first to present and evaluate a methodological approach to applying Peyton’s Four-Step Approach to skills-lab small group teaching. We described a modified Peyton’s Approach consisting of six defined parts, which was constructed by an expert team and was designed to be applicable to small group teaching. Results of the conducted evaluation of skills-lab training sessions on IV catheter insertion prove the practicability of the presented model for the setting described. The model was well accepted by trainees, and was rated as easy to realize, resulting in a good flow of teaching and success in attracting trainee’s attention when observed by expert raters. Qualitative semi-standardized interviews performed with all of the trainees and tutors revealed that learning by observation and repetition, learning by teaching, active involvement and the opportunity for independent performance made the modified Peyton’s approach a valuable learning experience.

Within the modified Peyton’s Approach, Steps 1 and 2 of Peyton’s Four-Step Approach are only performed once (Part I: “Demonstration and Deconstruction”), while during the “Circulation”, the modified Peyton’s Approach actively involves all participating students by delegating defined tasks (“Comprehension”, “Trainee’s Performance”, “Observation” and “Peer Feedback”). Performing Part I (“Demonstration and Deconstruction”) only once reduces the time needed and allows the most important steps of Peyton’s Four-Step Approach to be focused upon – namely Step 3 (“Comprehension”) and Step 4 (“Performance”), which are an integral part of the “Circulation”. Jawhari et al. [24] were able to show that Step 3 (“Comprehension”) of Peyton’s Four-Step Approach represents the central step within this approach, and is superior to a mere repetition of Step 2 (“Deconstruction”). Finally, Step 4 (“Performance”) is highly relevant, as the independent performance with subsequent feedback represents an indispensable prerequisite for subsequent deliberate practice. Within Part V (“Circulation”), the new model combines Step 3 (“Comprehension”) and Step 4 (“Performance”) of Peyton’s Four-Step Approach, as one trainee instructs another, instead of being instructed by the trainer, meaning that all of the trainees (except the last trainee) perform the task under verbal instruction. Beyond Peyton’s Four-Step Approach, the modified Peyton’s Approach incorporates peer feedback within Part V (“Circulation”), as feedback has proven to be highly relevant for learning success [5], and the verbalization of feedback itself might result in awareness of the correct procedure. This active involvement of all trainees seems to be of significant importance, as the defined tasks (comprehension, performance, observation and peer feedback) might have led to the fact that the expert ratings document that participants were highly focused.

Acceptance ratings provided by trainees revealed that they had the impression they had learnt a lot, and that they devoted all of their attention to the subject of the training. This was supported by expert ratings, which also revealed that tutors succeeded in realizing a good flow of training. Quantitative as well as qualitative analyses showed that trainees valued repeated observation, instruction of peers and the opportunity for independent performance, although they wished for more time for repeated practice, and individual students raised doubts about the transferability of trained skills to clinical settings. However, they felt well prepared for further independent practice, which reflects the didactic aim and background of the performed instructional approach. Further deliberate practice, as an essential part of skills-lab training [11], has to follow the performed instructional approach in order to reach mastery, as deliberate practice constitutes one of the most relevant factors for learning success in simulation-based medical education [5,37] and the presented instruction is only able to provide a prerequisite for deliberate practice. With regard to transferability, a study on IV cannulation training has demonstrated the transferability of skills-lab training in a clinical setting [9]. Tutors confirmed that trainees were highly focussed throughout the training and that they perceived the repeated observation as valuable preparation for their own performance. Tutors valued the learning success of trainees.

The described model reflects the attempt to preserve the relevant learning steps of Peyton’s Four-Step Approach, to propose a clear structure of instruction, to involve all participants and to focus and optimize the training. The problem of how to save as much valuable teaching and practice time as possible is well known in the cost-intensive field of simulation and skills-lab training. With the modified Peyton’s approach, teaching time can be halved compared to a 1:1 student : tutor instruction, as half the number of performances are needed. In the literature, several models to extract teaching steps that are not tutor-dependent and to realize an optimum of preparation of trainees are described. Sopka et al. [38] described a model to simplify Peyton’s Four-Step Approach when teaching basic life support, replacing Peyton’s Steps 1 and 2 with a self-produced podcast. Participants of both the intervention and the control group showed comparable performance even six months after the intervention. Lehmann et al. [39] used a blended learning approach with virtual patients to reach an optimum of preparation when finally starting the actual skills-lab training. These approaches are promising, although both of them afford financial expenditure and technical infrastructure.

The proposed modified Peyton’s Approach for skills-lab small group teaching in the current study was evaluated with groups of three trainees only. However, enlarging the group size might have led to more critical evaluations and reduced attention to the instruction, as Part V (“Circulation”) might have contained too many repetitions, inducing the feeling of having too little time for deliberate practice following the instruction.

Several limitations of the current study have to be mentioned. First, the sample size was rather small and only one skill was evaluated, potentially limiting the representativeness of the study and possibly leading to the themes within the qualitative analyses not being exhaustive. In line with this aspect, we evaluated the developed approach only with small groups consisting of three trainees each. The study did not address the realization of the approach within larger groups. Therefore, experiences with the approach have to be gained in larger groups, as skills-lab training is normally delivered with groups of four to eight students [4,25-28]. In this respect, it could be helpful to begin the instruction in a large group and to subsequently split the group into smaller groups to continue “Circulation” (Part V). It also remains unclear whether students benefit more from repeated observation and giving peer feedback or whether it would be preferable to begin earlier with their own independent performances in the sense of deliberate practice. Further studies should therefore seek to identify the time at which good preparation is reached before deliberate practice is started. Further limitations are to be seen in a potential self-selection bias due to volunteer recruitment, payment, usage of self-report scales and a lack of comparison with any other clinical skills (e.g. physical examination) to assess generalizability, and a lack of objective efficacy and long-term retention. Finally, it remains unclear what effect the presence of objective raters and supervisors had.

## Conclusion

In conclusion, the current study examined a new model based on Peyton’s Four-Step Approach to instruct small groups of students in skills-lab training sessions, which was revealed to be practicable, well accepted by trainees, and easy for tutors to realize. Further research should address the realization of the model in other settings and larger skills-lab training groups, and also research alternative instructional approaches.

## Competing interests

The authors declared that they have no competing interest.

## Authors’ contribution

CN conceived of the study, participated in the design of the study, analysed the data and drafted the manuscript. JH helped carrying out the qualitative analysis. JS participated in designing the study and helped to draft the manuscript. DH and JH helped designing and coordinating the study. WH participated in designing the study and helped to draft the manuscript. JJ participated in designing the study and helped to draft the manuscript. MK conceived of the study, participated in the design of the study, analysed the data and drafted the manuscript together with CN. All authors read and approved the final manuscript.

## Pre-publication history

The pre-publication history for this paper can be accessed here:

http://www.biomedcentral.com/1472-6920/14/68/prepub
